# Accuracy of the end-expiratory lung volume measured by the modified nitrogen washout/washin technique: a bench study

**DOI:** 10.1186/s12967-021-02703-x

**Published:** 2021-01-19

**Authors:** Joana Berger-Estilita, Matthias Haenggi, Daniel Ott, David Berger

**Affiliations:** 1 Department of Anaesthesia and Pain Medicine, Inselspital, Bern University Hospital, University of Bern, Bern, Switzerland; 2 Department of Intensive Care Medicine, Inselspital, Bern University Hospital, University of Bern, Bern, Switzerland; 3 Department of Radiology, Inselspital, Bern University Hospital, University of Bern, Bern, Switzerland

**Keywords:** Functional residual capacity, End-expiratory lung volume, Washin/washout technique

## Abstract

**Background:**

The functional residual capacity (FRC) determines the oxygenating capacity of the lung and is heavily affected in the clinical context of the acute respiratory distress syndrome. Nitrogen-wash-in/wash-out methods have been used to measure FRC. These methods have rarely been validated against exactly known volumes. The aim of the study was to assess the accuracy and precision of the N_2_ washout/washin method in measuring FRC, by comparing it with set volumes in a lung simulator.

**Methods:**

We conducted a diagnostic bench study in the Intensive Care Unit and Radiology Department of a tertiary hospital in Switzerland. Using a fully controllable high fidelity lung simulator (TestChest®), we set the functional residual capacity at 1500 ml, 2000 ml and 2500 ml and connected to the GE Carestation respirator, which includes the nitrogen washout/washin technique (INview™ tool). FRC was then set to vary by different levels of PEEP (5, 8, 12 and 15 cmH_2_O). The main outcome measures were bias and precision of the TestChest® when compared to the results from the washout/washin technique, according to the results of a Bland Altman Analysis. We verified our findings with volumetric computed tomography.

**Results:**

One hundred and thirty-five nitrogen-wash-in/wash-out measurements were taken at three levels of F_I_O_2_ (0.4, 0.5, 0.6). The CT volumetry reproduced the set end-expiratory volumes at the Simulator with a bias of 4 ml. The nitrogen-wash-in/wash-out method had a bias of 603 ml with acceptable limits of agreement (95% CI 252 to − 953 ml). Changes were detected with a concordance rate of 97%.

**Conclusions:**

We conclude that the TestChest® simulator is an accurate simulation tool, concerning the simulation of lung volumes. The nitrogen wash-in/wash out method correlated positively with FRC changes, despite a relatively large bias in absolute measurements. The reference volumes in the lung simulator verified with CT volumetry were very close to their expected values. The reason for the bias could not be determined.

## Background

Mechanical Ventilation is a life-saving method for patients with respiratory failure. ICU admissions requiring ventilator support range from 19 to 75% [1–3] and ventilator use is expected to increase further [4]. One of its most frequent applications is in patients with acute respiratory distress syndrome (ARDS). The damage caused by mechanical ventilation itself, due to the application of stress and strain to the lung [5], may potentiate ARDS further, creating a vicious cycle of disease. ARDS is associated with a marked reduction in lung volumes [6]: an estimate of the latter may therefore be fundamental for optimizing the ventilatory strategy and to minimize the ventilation-induced lung injury (VILI).

The functional residual capacity (FRC) is the amount of gas remaining in the lungs at the end of expiration during spontaneous breathing at atmospheric pressure [7]. In critically ill patients requiring mechanical ventilation, the level of PEEP determines FRC. The term end-expiratory lung volume (EELV) is used to indicate FRC, if PEEP is applied. The EELV represents the equilibrium point for the spring out forces of the chest wall and the collapsing tendency of the lung [8] and is a major determinant for the oxygenation capacity of the lung. Its knowledge and changes also allow for the determination of static lung strain (volume change divided by resting volume) and may help to improve ventilation. However, FRC measurements were, for several years, not routinely used in adult ICUs, due to technical reasons [9].

Olegard et al. [10, 11] have published a modified real-time, open circuit, multiple breath nitrogen wash-in wash out method, which allows calculating the end-expiratory lung volume without interruption of mechanical ventilation. This method only requires a step change in the inspired oxygen fraction (FiO_2_) instead of additional tracer gases. This tool is available as the FRC INview™ tool (GE Carestation, GE Healthcare, Chalfont St Giles, UK). It has been validated in ICU patients against CT-graphic volumetry [12] and helium dilution [13] with acceptable accuracy. The only validation against a known volume was done by the describers of the method [14], lacking further independent confirmation, which was the aim of this study.

## Methods

The manuscript adheres to the applicable STARD (Standards for Reporting Diagnostic accuracy studies) Guidelines. (See Additional file 1: STARD-2015-Checklist.docx)

We performed a prospective bench study using a lung simulator (TestChest®, ORGANIS GmbH, Switzerland) to assess the accuracy of the GE Carestation and its INview™ tool for FRC determinations. The TestChest® allows to set the end-expiratory volume. We validated the set values for FRC at the TestChest® against computed tomography (CT) scanning, taken as the reference gold standard [12, 15]. This volumetric step was necessary since the precision of the mechanical bellows was unknown and respective data could not be gathered at the manufacturer. After validating the TestChest, we used the TestChest® simulator to validate the nitrogen-washin/washout.

We used a TestChest® high-fidelity lung Simulator (ORGANIS GmbH, Switzerland, Software version 2.06) as a reference tool for the end-expiratory lung volume. The TestChest® is a full physiologic artificial lung that can simulate gas exchange and hemodynamic responses of the healthy and pathological adult lung [16]. It has a programmable Functional Residual Capacity (FRC) and allows the simulation of lung collapse and recruitment as well as hysteresis of the pressure–volume loop. Carbon dioxide production (VCO_2_) is simulated with the use of a mass flow controller. The device was calibrated according to the manufactures description. Three functional residual capacities (1500 ml, 2000 ml, 2500 ml) were set on the TestChest® at a stable respiratory systems compliance of 60 ml/cmH_2_O and a VCO_2_ of 240 ml/min in standard temperature and pressure, dry (STPD) conditions. The simulated cardiac output was set at 7000 ml/min. The device does not have an oxygen consumption cell.

We used the nitrogen washout/washin technique (INview™ tool) as implemented by the GE Carestation [10]. The ventilator was connected to the lung simulator by a conventional circuit hose (22 mm diameter, 1.6 m length, Flextube Intersurgical, Germany) with separate inspiratory and expiratory limbs with the use of a heat–moisture exchanger (HME). Prior to each measurement, the ventilator (including the nitrogen analyzer) was calibrated and tested for leaks.

The three functional residual capacities (1500 ml, 2000 ml, 2500 ml) that were set on the TestChest® were measured by the N_2_ washin/washout open circuit method [11]. Measurements were initially taken at zero PEEP. Then, EELV was modified by PEEP of 5, 8, 12 and 15 cmH_2_O. All measurements were repeated at fractions of inspired oxygen (FiO_2_) of 0.4, 0.5 and 0.6, summing up to a total of 135 measurements. The ventilator was set in a volume controlled mode at a tidal volume of 500 ml and a respiratory rate of 15 breaths per minute.

As the TestChest® does not simulate a VO_2_, a possible error was calculated based on an assumed respiratory exchange ratio of 0.8, the VCO_2_ set at 240 ml/min would correspond to a VO_2_ of 300 ml/min. This VO_2_ has to equal the oxygen uptake from the ventilator, according to the formula below

$$VO_{2} = MVx(F_{i} O_{2} - F_{e} O_{2} )$$

where MV is minute ventilation and FiO_2_ and FeO_2_ are the in- and expired fractions of oxygen.

### CT Scan data collection

**Fig. 1:**
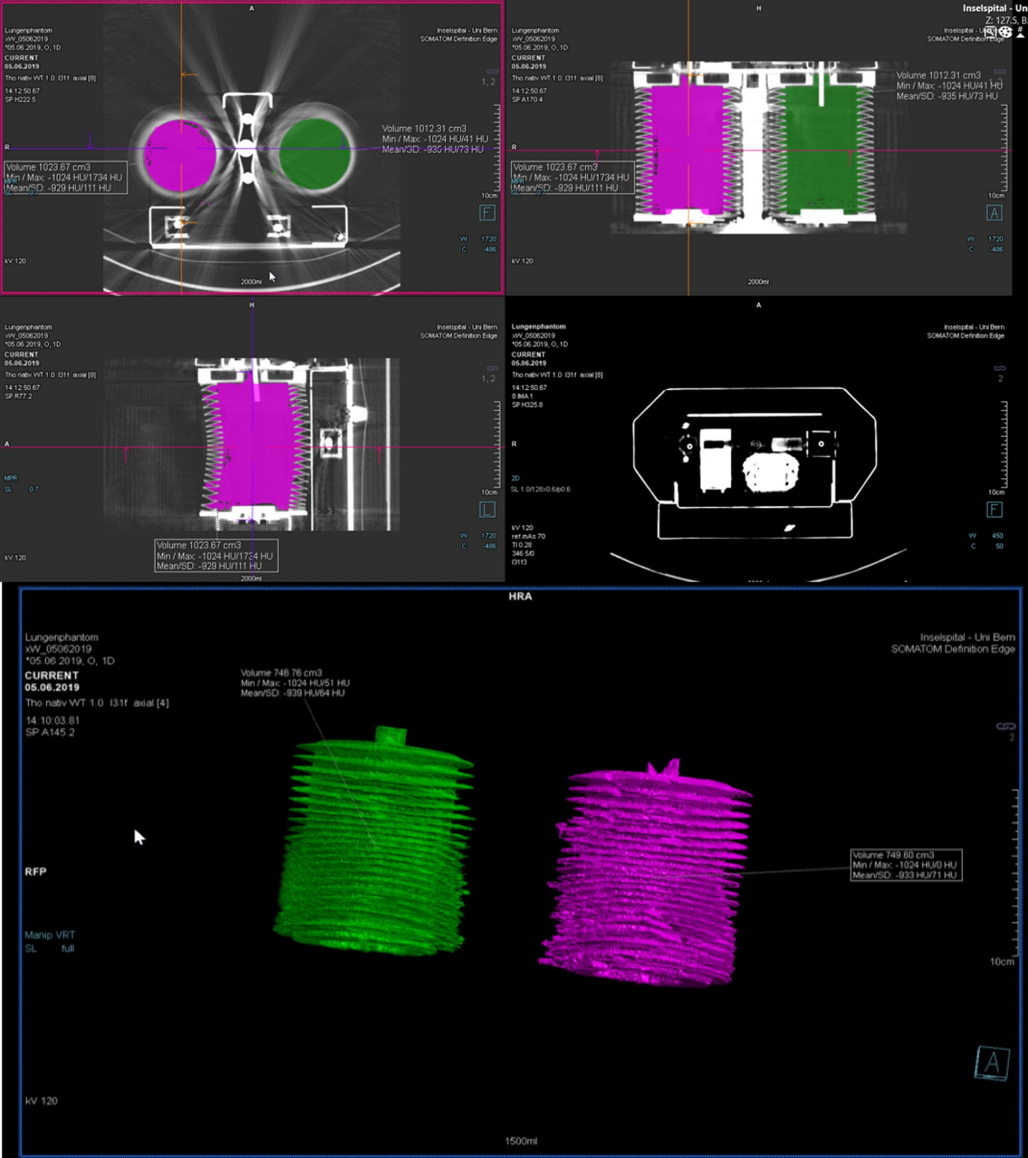
An example of a CT Volumetry at a set endexpiratory lung volume of 1500 ml is shown

Images were acquired on a third-generation Single-Source CT (Somatom Definition Edge; Siemens Healthineers, Forchheim, Germany). Computed tomography scans were performed with a tube voltage from 100 to 120 kVp and a reference mAs of 70. On the 128-detector scanner, a collimation of 128 × 0.6 mm was used, with a pitch of 0.6. A slice thickness of 1 mm was reconstructed, with a SAFIRE (Sinogram Affirmed Iterative Reconstruction) level 3 and with a soft tissue kernel of I31f. Then the “Lungs” (bellows) were manually segmented using SyngoVia (Version VB30A), a Post-Processing-Platform (Siemens Healthineers, Forchheim, Germany), to calculate the Volumes. An exemplary picture is given as Fig. 1.

### Statistical analysis

Statistics were performed with SPSS Software Version 25 (IBM Coorporation, SA). Graphics were produced with SigmaPlot 14.0 (Systat Software GmbH, Erkraht, Germany). The agreement between set and measured FRC gas volumes measured with the nitrogen washout/washin technique was analyzed with a Bland & Altman (B–A) analysis [17]. EELV at different FiO_2_ levels were compared with a two-way mixed effects intra-class correlation for absolute agreement [18] and a one-way ANOVA for repeated measurements. A p-value of < 0.05 was considered significant. Data is presented as mean ± SD.

## Results

### CT volumetry

The CT volumetry reproduced the set end-expiratory volumes at the TestChest (1496 ml, 2035 ml, 2458 ml, r2 0.995, p < 0.044) with a bias of only 4 ml on average.

### Overall performance of end-expiratory lung volume measurements

**Fig. 2:**
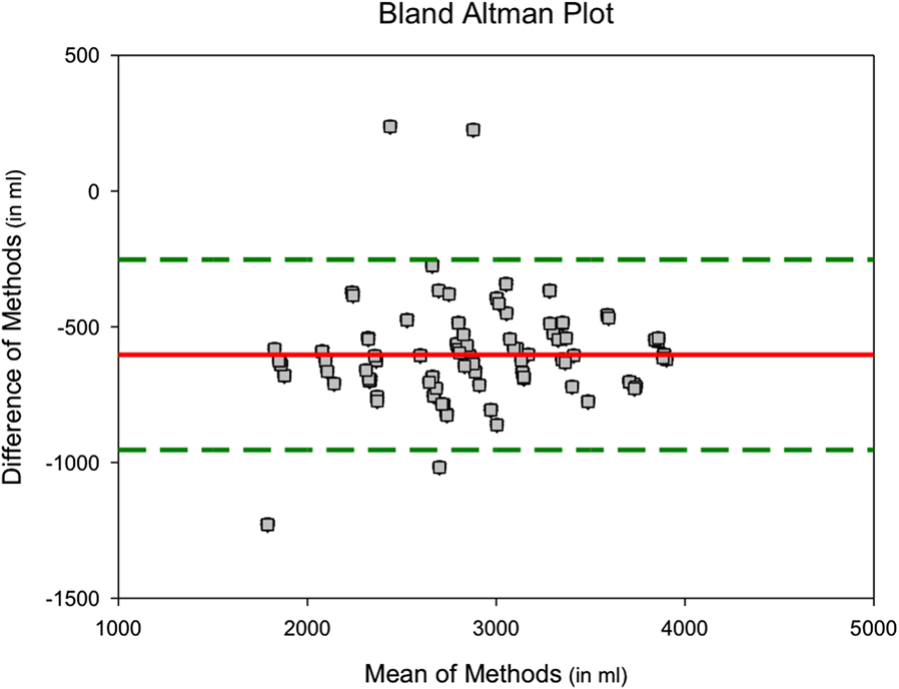
Bland Altman Plot of Bias and Precision between the nitrogen wash-in/wash-out method and the set volumes at the TestChest®

All 135 planned measurements were performed. There was a strong, positive correlation (r^2^ = 0.909, EELV_N2_ = 762 ml + 0.936 * EELV_TestChest®)_ between the end-expiratory lung volume measured with the modified nitrogen wash-in/wash-out and the set volumes on the TestChest®. The bias was 603 ml towards an overestimation of the EELV with the N_2_ method with acceptable limits of agreement (252 to − 953 ml) (Fig. 2). There is no correlation between the means and differences in the Bland–Altman Analysis (r^2^ 0.02, p = 0.13).

### Influence of FiO_2_ on the measurements

The measurements over the range of 40, 50 and 60 percent of oxygen were 2425 ± 136 ml, 2409 ± 146 ml, 2419 ± 142 ml, statistically different (p < 0.001), but of a clinically irrelevant magnitude. The intraclass correlation between the FiO_2_ levels was excellent (0.980, 95% Confidence Interval 0.964 to 0.989, p < 0.001) [18].

### Trend and changing EELV

**Fig. 3:**
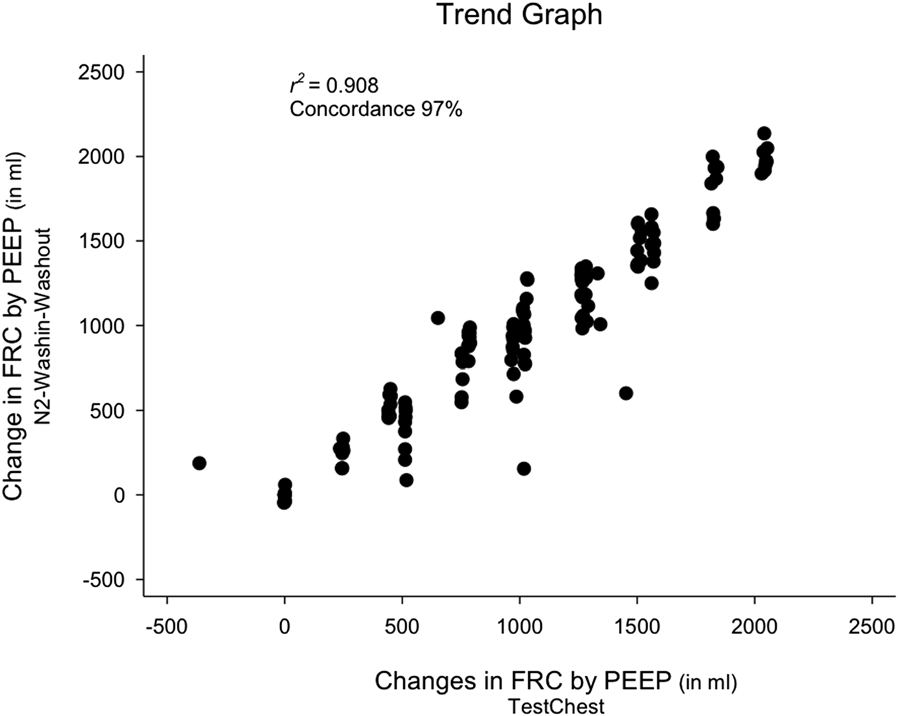
Four-Quadrant Plot for the determination of trending abilities. Changes in Volume induced by PEEP are plotted (on x-axis) versus the measured changes in volume on the y-axis. The regression equation for changes in measured volume is 27.025 ml + 0.937 * change in set volume

The changes in end-expiratory volume induced by changes in PEEP were tracked very well. The concordance rate and the correlation were excellent (Fig. 3).

### Calculation of the error attributed to VO_2_

The oxygen uptake from the inspired air was calculated to be 4% of total volume and was stable at every FiO_2_. This means that the FeO_2_ would be 4% lower than the FiO_2_, if an oxygen burning cell would have been in place. This error leads to a 4% additional nitrogen washout of total applied volume. (See Additional file 2: Stepwise calculation of the error introduced by the lack of an oxygen burning cell).

## Discussion

For FRC determination, our results show an excellent agreement of the end-expiratory volumes set on the TestChest®, when compared with the gold standard method (CT volumetry). This made it possible to use the TestChest® as a reference volume. Additionally, we demonstrated the accuracy of the TestChest® for the correct simulation of static lung volumes. This may make it an apparently reliable tool for validating volumetric measurement devices. Finally, the FRC determination using the modified N_2_-Washout/Washin method showed a bias of overestimation of 603 ml. There was a clinically irrelevant influence of the step change of FiO_2_ on the measurements and a small correction for O_2_ consumption.

The FRC gives information about the chest wall/lung system because it represents the equilibrium point for the forces of the chest wall and lung and it is a valuable tool for optimizing respiratory settings [9, 19–22]. Still, it tends to be sidelined behind other parameters, most probably because of technical difficulties in its determination.

The N_2_-washout/washin technique, initially developed in the beginning of the 1900’s [23], never really gained popularity because of the cumbersome use of mass spectrometry. A later development [24], which relied on O_2_ and CO_2_ measurements, needed an unrealistic (and unsafe) step change of 30% in the FiO_2_ and also a very sensitive flow—gas synchronization. The modified technique from Olegard [10] overcame these limitations by requiring only the small change (10%) of FiO_2_, without the need for special gas analysers. However, it still showed some degree of FRC overestimation in in vivo settings [10, 12]. Finally, since the EELV relies on a measure of ventilated lung, such measurements can be underestimated in several disease states and in patients with poorly ventilated lung compartments. This is a limitation that may be overcome by the use of a lung simulator.

The TestChest® allows the choice and setting of various parameters of lung mechanics and gas exchange. As such, it may be a valuable tool for teaching and evaluating the performance of devices for physiological measurements. But little is known about its accuracy. The end-expiratory lung volumes that we had set at zero PEEP were almost identical with the CT volumetry. We could, therefore, demonstrate accuracy of the TestChest® for the correct simulation of static lung volumes.

We could also demonstrate that the nitrogen washout/washin technique showed a good correlation with the TestChest®, indicating that the trend of the FRC is trustworthy and can be used in clinical settings. But when we verified the volumes and modified them with the application of PEEP, our results were not in agreement with previous published work. To our knowledge, only one other study [12] compared the nitrogen washin/washout method with the CT scan FRC estimation, in ICU patients. In that study [12], the measurements between the nitrogen washin/washout method and the EELV computed by the CT scan showed a high correlation and a bias of only 100 ml, about 5% of the FRC, when estimated by the formula recommended by the European Respiratory Society [25]. In our case, the bias is much greater, reaching approximately 600 ml, up to one third of the set FRC. While we could reproduce the good correlation between the two measurements, this bias is intriguing. The overestimation of FRC is known for in vivo settings [10, 12], but not in this magnitude. Additionally, the bias is very stable over the measured range of volumes. The TestChest® lacks an oxygen consumption cell. This will lead to an additional 4% of expired oxygen, which artificially dilutes the expired N_2_, falsely indicating retained nitrogen in the lung. Still, our bias exceeds the expected four percent error by far.

In our setting, the breathing circuit hose had a diameter of 22 mm and a length of 1.6 m, reflecting a volume of 608 ml, which is very close to the given bias. Since gas measurements take place within the ventilator unit and not at the y-piece, apparatus dead space may contribute to the increases in EELV with the N_2_ method. A third explanation would be incomplete gas mixing in the bellows and a contribution of what is known as the “first-breath problem” [11, 24]. All these explanations are supported by the fact that the bias stays constant over a wide range of measured volumes, therefore representing an artifact of the methodology. Finally, as the TestChest® simulator was set at STPD conditions, the lack of humidified expired air may have introduced a diluting effect on the expired nitrogen. However, our data is the only comparison of the open circuit N_2_ method with a known volume besides the initial simulation data of Olegard [14].

It has been argued that the absolute value of the FRC is of less interest than its trend during the course of disease or after, for example, a recruitment maneuver [26]. This implies that accuracy is less important than reproducibility. Despite the large bias, changes in end-expiratory volume were tracked very accurately by the N_2_-washin-washout. The different settings of FiO_2_ did not influence the accuracy of the measurements.

## Conclusion

In conclusion, the EELV set at the TestChest® is highly accurate compared to the gold standard of a CT scan. When using it as a validating device for dilutional methods, the lack of an oxygen consumption cell or incomplete gas mixing in the bellows may contribute to bias.

We conclude that the TestChesT® simulator is an accurate simulation tool, concerning the simulation of lung volumes. It may serve as a reference tool for validation of static lung volumes.

## Supplementary Information

**Additional file 1.** STARD-2015-Checklist.

**Additional file 2.** Stepwise calculation of the error introduced by the lack of an oxygen burning cell.

## Data Availability

All data generated or analysed during this study are included in this published article and its additional information files.

## Abbreviations

ARDS: Acute respiratory distress syndrome
B-A: Bland & Altman
CT: Computed tomography
EELV: End-expiratory lung volume
FRC: Functional residual capacity
STARD: Standards for Reporting Diagnostic accuracy studies
STDP: Standard temperature and pressure, dry
VCO_2_: Carbon dioxide production
VILI: Ventilation-induced lung injury
