# Supplementary material for: Accuracy of the end-expiratory lung volume measured by the modified nitrogen washout/washin technique: a bench study
Source: J Transl Med. 2021 Jan 19;19:36. doi: 10.1186/s12967-021-02703-x (PMC7815189; doi:10.1186/s12967-021-02703-x)
Supplement: Supplementary file 2 — Additional file 2. Stepwise calculation of the error introduced by the lack of an oxygen burning cell. [file 12967_2021_2703_MOESM2_ESM.docx]

*Stepwise calculation of the error introduced by the lack of an oxygen burning cell*

The numeric calculation can be found in the excel sheet attached. At the TestChest, a VCO_2_ of 240 ml/min was set. A VO_2_ cannot be directly simulated. By assuming a respiratory exchange ratio, the corresponding VO_2_ can be calculated (Formula 1 and 2).

$RER= \frac{{VCO}_{2}}{{VO}_{2}}$ (1)

${VO}_{2}=\frac{{VCO}_{2}}{RER}$(2)

For 240 ml/min of carbon dioxide produced and a normal RER of 0.8, the VO_2_ would correspond to 300 ml/min. This oxygen needs to be extracted from the inspired gas mixture. F_E_O_2_ can therefore be calculated according to formula 3 and 4 (MV=Minute Ventilation).

$${VO}_{2}=MV\left( F_{I}O_{2}-F_{E}O_{2} \right)(3)$$

$$F_{E}O_{2}=F_{I}O_{2}-\frac{{VO}_{2}}{MV} (4)$$

For our given example this corresponds to an in- to expiratory oxygen fraction difference ΔFO_2_ of 4%. FeCO2 can be calculated similarly (Formula 5), whereby FiCO_2_ is zero.

$$F_{E}{CO}_{2}=F_{I}{CO}_{2}-\frac{{VCO}_{2}}{MV} (4)$$

Numerically, FeCO_2_ is 3.2% in our example, which is 80% of the FeO_2_, according to the RER.

Inspired Nitrogen can be calculated as either volume per time or fraction:

${V_{I}N}_{2}=MV\left( 100\%-F_{I}O_{2} \right)$ (5)

${F_{I}N}_{2}=100\%-F_{I}O_{2}$(6)

Expired Nitrogen Fraction be calculated a follows with Fe_total_ = 100%:

$${F_{E}N}_{2}={F_{E}}_{total}-{F_{E}CO}_{2}-{F_{E}O}_{2}(7)$$

The test chest does not consume oxygen, so we must assume FiO_2_ = FeO_2_. There are now two solutions to equation 7. One in the TestChest® (F_I_O_2_ = F_E_O_2_), and one with a real VO_2_ and F_I_O_2_ > F_E_O_2_.

| **Testchest (F_I_O_2_ = F_E_O_2_),** | **Reality (F_I_O_2_ > F_E_O_2_)** |
| --- | --- |
| ${F_{I}N}_{2}={F_{I}}_{total}-F_{I}O_{2}$ | ${F_{I}N}_{2}={F_{I}}_{total}-F_{I}O_{2}$ |
| ${F_{E}N}_{2}={F_{E}}_{total}-{F_{E}CO}_{2}-{F_{\boldsymbol{I}}O}_{2}$ | ${F_{E}N}_{2}={F_{E}}_{total}-{F_{E}CO}_{2}-{F_{\boldsymbol{E}}O}_{2}$ |
| ${F_{I-E}N}_{2}={F_{E}}_{total}-{F_{E}CO}_{2}-{F_{\boldsymbol{I}}O}_{2}={F_{E}CO}_{2}$ | ${F_{I-E}N}_{2}={F_{E}}_{total}-{F_{E}CO}_{2}-{FO}_{2}={F_{E}CO}_{2}+{FO}_{2}$ |

This means that the exhaled nitrogen in the TestChest® is lower by the amount the oxygen extraction from expired air than if there was oxygen consumption. This artificial “dilution” of the exhaled nitrogen will be misinterpreted as additional nitrogen retention, leading to falsely high end-expiratory lung volumes. Note that the relative error will increase with increasing FiO_2_ and that within a step change of 10% of FiO_2_ for the measurement, a 4% error in gas fraction is very substantial.

Note that the TestChest® works at STPD conditions.
